# What Are the Participants' Perspectives of Taking Melatonin for the Treatment of Nocturia in Multiple Sclerosis? A Qualitative Study Embedded within a Double-Blind RCT

**DOI:** 10.1155/2018/4721505

**Published:** 2018-10-18

**Authors:** Rafiyah Khan, Alan Uren, Luke Canham, David Cottrell, Marcus J. Drake, Nikki Cotterill

**Affiliations:** ^1^Bristol Urological Institute, UK; ^2^Neurology Department, North Bristol NHS Trust, UK; ^3^Translational Health Sciences, University of Bristol and Bristol Urological Institute, UK

## Abstract

**Background:**

Multiple Sclerosis (MS) is a chronic neurological disorder caused by neurodegeneration within the central nervous system. It results in impaired physical, cognitive, and psychological functioning and can also lead to lower urinary tract symptoms including nocturia. While clinical trials have suggested an association between nocturia and melatonin secretion, to our knowledge, no qualitative research has been conducted on the experience of taking melatonin to treat nocturia in progressive MS within a clinical trial.

**Methods:**

17 semistructured qualitative interviews were conducted as part of a double-blind, randomised, placebo controlled, crossover, clinical trial with consenting adults with MS. Interviews explored participants' experiences of nocturia associated with MS and their experience of taking melatonin as a trial treatment for nocturia versus a placebo. Data was analysed using a thematic analysis.

**Results:**

Themes on the experience of nocturia revealed participants' understandings of nocturia, the impact it had on their night, and increased daily fatigue. Themes on the intervention showed perceived improvements to nocturia, sleep, and energy and negative effects including lethargy, a lack of significant change, and physical side effects including vivid dreams.

**Conclusion:**

This qualitative exploration revealed an association between nocturia and increased levels of fatigue during the day by those with MS. However, perspectives towards the effectiveness of melatonin as a potential treatment varied as both placebo and melatonin were perceived as having very similar effects.

## 1. Introduction

Multiple Sclerosis (MS) is a chronic neurological disease which affects over 100,000 people in the UK [1]. It is characterised by progressive neuronal degeneration secondary to neuroinflammation within the central nervous system often resulting in impaired physical, cognitive, and psychological functioning and the presentation of a range of different, unpredictable symptoms. Such neurodegenerative diseases are also known to damage the neuronal pathways involved in lower urinary tract control [2]. As a result, people with MS often report a range of lower urinary tract symptoms (LUTS) including hesitancy, retention, urgency, incontinence, frequent urination, and nocturia [3, 4].

Nocturia is a common condition and is defined by the International Continence Society (ICS) as the “need to wake one or more times at night to void” [5]. It is frequently cited as one of the most bothersome urinary symptoms and can have a significantly adverse effect on quality of life [6]. This perception often increases with the number of sleep disturbances per night [7]. For those with MS, consistently waking up to pass urine during the night can be a significant concern especially when suffering from advanced disease. In such situations, physically getting up to urinate can be a physical challenge in itself, leading to falls and potentially disrupting the sleep of their carers and/or family member.

Disrupted sleep can also exacerbate the experience of fatigue during the day. This is particularly important as fatigue is considered one of the most troublesome MS symptoms and can have a detrimental effect on quality of life [7]. It interferes with an individual's ability to partake in many daily activities and is also closely correlated with depression [8]. Though the causes of MS related fatigue are relatively unknown, sleep disturbances such as nocturia undoubtedly contribute to its severity. Positive correlations have been found between waking during the night and the presences of fatigue during the day and respondents identified nocturia as a key contributing factor [9].

Melatonin, a hormone secreted primarily at night, regulates circadian rhythms and reduces spontaneous smooth muscle activity including that in the bladder [10]. Melatonin secretion can be impaired in MS and it was hypothesised that its administration could improve nocturia among individuals with MS [11]. It has been evaluated in elderly people and men as a potential treatment for nocturia [12, 13]. The present double-blind, randomised, placebo controlled, crossover clinical trial sought to extend these findings to individuals with MS by assessing the effectiveness of melatonin on the number of nocturia episodes per night. The current report describes the qualitative phase of the study with a particular focus on the lived experience of nocturia from the perspective of adults with progressive MS, and where possible their partners. It will also discuss the experience of taking melatonin and a placebo for the treatment of nocturia while participating in the trial.

## 2. Materials and Methods

### 2.1. Overview

This qualitative exploration was embedded within a double-blind, placebo controlled, randomised controlled, crossover trial of melatonin for the treatment of nocturia in adults with Multiple Sclerosis (MeNiMS) [11]. This consisted of two treatment periods which involved administering the intervention drug melatonin for six weeks and the placebo for a further six weeks with a one month wash out period between each treatment phase. The order of administration was randomised and consenting participants were given 2mg per night of sustained-release melatonin (Circadin) or a placebo capsule, identical in appearance to Circadin, per night. During the trial, participants were invited to take part in five study visits to monitor progress and were interviewed by a qualitative researcher following their final study visit. This was approved by the National South West Research Ethics Committee, Exeter, UK (REC reference number: 12/SW/0322).

### 2.2. Participants

Patients with MS were recruited to the MeNiMS trial from North Bristol NHS Trust with the following inclusion criteria: (1) aged over 18, (2) having diagnosed MS according to McDonald criteria, (3) having at least one episode of nocturia per night as defined by ICS, and (4) female subjects of childbearing potential having to be willing to use an effective method of contraception throughout the study. Eighteen participants from the total MeNiMS trial population were approached for the qualitative interview and seventeen provided written consent. The sample consisted of ten female participants and seven males with an age range of 39 to 67 (mean: 58 years). Six patients had Relapsing Remitting MS (RRMS), two Primary Progressive MS (PPMS), and nine Secondary Progressive MS (SPMS). The sociodemographic characteristics of participants are presented in Table 1. Clinical quantitative data regarding EDSS (Expanded Disability Status Scale), quality of life associated with MS and nocturia, bladder dysfunction, and sleep quality evaluation are detailed by treatment groups in the primary trial outcome paper [14].

### 2.3. Data Collection

#### 2.3.1. Semistructured Interviews

Prior to their final study visit, consenting participants were contacted by the qualitative research team via telephone and were given the opportunity to discuss the qualitative study. In total, seventeen semistructured face to face interviews were conducted and where possible, the partners of participants were also involved in these discussions. Interviews took between 20 and 40 minutes to complete and were facilitated by a topic guide (Box 1) that was specifically designed to elicit an exploration of the following qualitative research aims:

(1) Explore patients' (and partners') perspectives of nocturia associated with progressive MS.

(2) Explore perceptions of the intervention on nocturia and associated quality of life impact from the patients' (and partners') perspective.

### 2.4. Thematic Analysis

All interviews were audio recorded and the data was transcribed verbatim. Transcripts were then coded using NVivo 10 and analysed using a thematic analysis [15]. Based on recommendations by Braun and Clarke [15], an inductive approach to analysis was incorporated. Transcripts were read and reread multiple times to allow an in depth familiarisation with the data. Important aspects of the data were then identified and coded descriptively and synthesised into themes. Finally, the first author was unblinded and themes were reviewed in light of the order of treatment to ensure an accurate understanding of participants' experiences regarding the effectiveness of melatonin. All themes were divided into two key concepts which reflected both research questions.

## 3. Results

**Figure 1:**
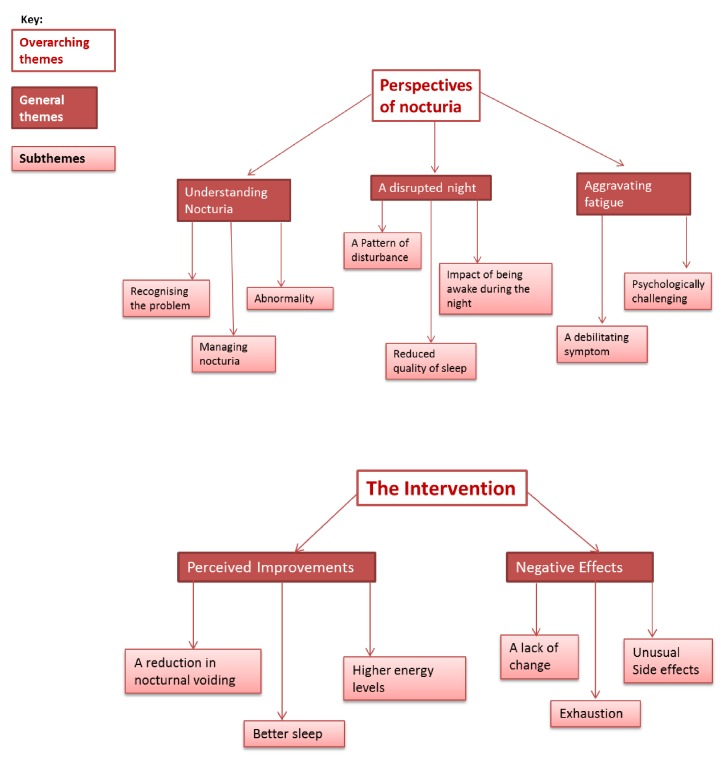
Summary of qualitative themes and subthemes.

A total of five themes and fourteen subthemes were found (Figure 1) and grouped into two key categories to reflect the research questions.

These categories were “participants” perspectives of nocturia' and “participants” perceptions of the intervention'.

### 3.1. Part 1: Perspectives of Nocturia

Three overarching themes emerged in relation to participant's perspectives of nocturia and their experience of this urinary symptom: (a) understanding nocturia, (b) a disrupted night, and (c) aggravated fatigue. Further to this, a number of interconnected subthemes were also explored.

#### 3.1.1. Theme 1: Understanding Nocturia

*Recognising the Problem*. Several participants had struggled to recall the precise onset of nocturia and described it as a progressive, gradual symptom that had increased in severity in recent years. Some participants viewed it as a sign of their transition into older age; a view that was strengthened by its prevalence within social circles. Participants also emphasised the role of the trial in enabling them to develop a better understanding of this relatively unknown symptom.

“You think it's not just me then, everybody seems to be getting up in the middle of the night” (Participant 022)

“I suppose still in the back of my mind I was thinking this is going to clear up it was only when they suggested going on the trial and now I think well there is a problem” (Participant 032)

*Managing Nocturia*. Nocturia was perceived as another symptom which heightened the challenging nature of MS and fatigue. Thus, participants stressed the importance of preventing the symptom from further hindering their daily activities. Individuals incorporated various management techniques into their lives (e.g., restricting their liquid intake) and it was noted that trial participation resulted in a better understanding of their nocturia which assisted in the development of coping strategies.

“It gets too urgent and then you can't control yourself so you have to go but your body's forcing you out of bed no matter what you do” (Participant 001)

“I think most people get up once or twice and it's manageable isn't it but it's not manageable when it's five, six times a night” (Participant 005)

*Abnormality*. Participants described their gradual realisation that their need to wake up to pass urine was problematic. For participants, nocturia had gradually worsened resulting in negative daily consequences. Moreover, the symptom was increasingly associated with a feeling of being different; an already prevalent feature of their life. It was noted that it was a desire for “normality” that had motivated them to participate in the trial and treat nocturia.

“My husband doesn't get up during the night, friends, if I'm on holiday with them they don't and it's like how come I do?” (Participant 029)

#### 3.1.2. Theme 2: A Disrupted Night

*A Pattern of Disturbance*. Nocturia was described as a regular occurrence and would often occur at consistent times during the night. This varied between participants but an individual pattern was commonly reported with most participants agreeing that the initial disturbance would take place a few hours following initiation of sleep. Participants had felt that the disturbances would coincide with their attempts to fully engage in deep sleep.

“It was happening at half past four in the morning every night and I thought well that is a problem because that's when you should be deeply in sleep” (Participant 023)

*Reduced Quality of Sleep*. All participants reported a broken, disrupted sleep experience which contributed to an overall sentiment that nocturia had substantially interfered with the quality of sleep. Both partners and participants provided detailed descriptions of poor sleep and estimated that waking to pass urine reduced their sleeping hours.

“I'm getting up four times a night I mean that's four hours of my total sleep gone” (Participant 030)

“It's so rare that he really gets a good night's sleep; I don't think there are any nights that you don't get up at all” (Partner of Participant 001)

*Impact of Being Awake during the Night*. Participants detailed their experience of being awake during the night due to nocturia. Urinary hesitancy, urgency, and incomplete emptying meant that passing urine during the night was often a drawn-out experience. Participants with mobility issues specified that this could potentially result in injury as participants felt disoriented when awake in the night. Almost all participants recalled anxiety and frustration as they began to anticipate a negative daily impact. Some had also incorporated distraction methods to initiate sleep such as meditation, reading books or watching television which was also used as a mechanism to prevent disruptions to their partners' sleep. This was a specific concern, as some participants explicitly described the guilt and embarrassment that they felt as they recalled disturbing others.

“Every time I'd even gone away to the loo and came back and got into bed I felt like I wanted to go again. It was really, really annoying” (Participant 005)

“I mean he [partner] is a shift worker so he doesn't get enough sleep sometimes anyway because of his shift patterns and for me then to disturb him, I don't like doing that it's not fair” (Participant 004)

“I think I'm quite a light sleeper so [021]'s quality of sleep generally affects mine” (Partner of Participant 021)

#### 3.1.3. Theme 3: Aggravating Fatigue

*A Debilitating Symptom*. Of all the symptoms reported by participants, fatigue was considered the most debilitating and was interlinked to the tiredness caused by nocturia. As their day began, participants described overwhelming feelings of tiredness which were primarily attributed to nocturia. They lacked energy and motivation and this was a barrier to maintaining their day to day lives. Those in employment required flexible working hours as waking up at certain times was difficult. Some would participate in shift work, drink caffeine when necessary or sleep in short periods throughout the day. Fatigue also negatively affected the interpersonal lives of participants as they struggled to interact in social situations.

“Fatigue, that's the issue but that's caused sometimes because I'm not getting the right amount of sleep as well which doesn't help so it's the two together really” (Participant 004)

“It's a pain, it makes you worse the following day you never have the energy you're more irritable and you don't have the energy to function in the way that you'd like to function like probably most people do by not getting up to the loo all night long” (Participant 002)

*Psychologically Challenging*. Participants also described the substantial psychological effect as they reported feelings of anxiety and embarrassment. They concluded that the combination of fatigue and severe tiredness resulted in anxiety, depression, low mood and irritability. This consequently affected motivation as participants expressed a fear of partaking in new activities. Participants also felt that it caused cognitive difficulties resulting in embarrassment.

“I think the worse thing with MS is the frustration because you want to do something and that you're just limited because of your energy levels” (Participant 033)

“If I have a bad night's sleep and I've got an appointment in the morning then that creates another level of anxiety because I know that if I don't process everything properly then it could be very, very embarrassing” (Participant 022)

### 3.2. Part 2: The Intervention

Data that emerged during discussions on the effectiveness of the intervention was coded into two key themes that aptly captured the participants' perspectives. All participants essentially summarised positive and negative experiences and as such all themes were grouped into either “perceived improvements” or “adverse effects” accompanied by subthemes.

#### 3.2.1. Theme 1: Perceived Improvements

*Reduction in Nocturnal Voiding*. A total of nine participants reported a reduction in nocturnal voiding when taking melatonin. However, one respondent specified that this had been a temporary improvement and their outcome measurements showed they experienced a more substantial improvement while taking the placebo. In fact, four respondents described urinary improvement when taking placebo. The perceived reductions ranged from twice nightly to a total reduction in nocturnal voiding. These changes were viewed as positive if they enabled participants to better manage their sleep.

“This is brilliant but after about four, five or six days they came back and then it was once a night” (Participant 022 – placebo)

“Because it's once or twice you can then get back to bed … you suddenly go back to sleep” (Participant 002 - melatonin)

*Better Sleep*. Improvements to the subjective experience of sleep were specifically described by six participants while taking melatonin. A further two participants noted that melatonin had helped induce a feeling of restfulness that resulted in a deeper sleep and made initiating sleep much easier. Two participants had also discussed this change while taking a placebo. For the participants who had reported fewer incidences of nocturnal voiding during the night, the lack of disruptions allowed them to engage in deeper sleep for a longer period.

“I'm dropping off to sleep easier [than] before and I am also going back to bed and going to sleep, it's quite a big improvement. I feel better, I don't feel so foggy” (Patient 032 - melatonin)

“From memory I didn't get up at all but as I said when I did I could go to the loo come back and think right I'm just going to go straight to sleep now and I did and that was good” (Patient 004 - melatonin)

“The quality of sleep is a lot better and because the quality of sleep's a lot better I feel a lot better I've got more energy I just felt great” (Patient 030 – placebo)

*Higher Energy Levels*. Most participants who had reported positive changes to sleep also articulated that this had caused significant improvements to their emotional state and they recalled a better mood, more enthusiasm throughout the day and a significant increase in perceived energy levels and mental alertness. This resulted in positive interpersonal changes that were visibly noticeable as two participants also recalled the positive feedback that they received from family members while taking melatonin. In total, these changes were reported by six participants during the melatonin phase and three while taking placebo.

“He's happier when he wakes up; it makes a difference in the day” (Partner of Patient 002 - melatonin)

“Brilliant… more va va voom, gives you more energy I suppose from not constantly being interrupted going to the toilet during the night and solid sleep which is lovely” (029 - placebo)

#### 3.2.2. Theme 2: Negative Effects

*A Lack of Change*. For respondents, one phase was often characterised by a continuation of their pretrial symptoms and the common belief was that this was the placebo. Participants reporting this during the first part of the trial remained hopeful that the second drug would be far more effective and would result in the improvements that they desired. However, those who had experienced positive changes when taking melatonin during the first phase reported a slow progressive decline in improvements during their placebo phase. They specified that it was during the final week of this phase that their pretrial symptoms had completely returned. Three participants also reported that neither drug had been particularly effective.

“These last lot I've had over the last four weeks I've been awake quite often during the night and they've just done nothing to be honest with you I haven't noticed anything” (Patient 004 – placebo)

“I could have been on the placebo both times really, I didn't notice enough to go wow that's made a difference” (Patient 003 – both phases)

*Exhaustion*. For seven participants, the trial had been particularly challenging as they experienced exhaustion, tiredness and an increased desire to sleep. For some this was more severe, while others described it as a slight increase in tiredness. Five of these reports were relayed by participants when taking melatonin which was thought to have induced a feeling of sleepiness that made it much more difficult for these participants to wake up in the morning. This tiredness continued throughout the day and was described in different ways, most commonly as drowsiness. This intensified the negative emotions reported by participants who felt more irritable.

“I found it hard to come round in the mornings, I don't think I went off to sleep much quicker may be a bit but it was really in the mornings that I tend to notice the thick headedness and difficulty waking up” (Patient 027 - melatonin)

‘I felt more lacklustre and lost energy on the second drug than I normally did' (Participant 021 – Placebo)

“The last three or four weeks I've been very irritable… my concentration's gone. I can't seem to want to read, I normally read and I don't read so much. I've got no interest in films… very little I like to do now that I get enjoyment from… It doesn't seem to be to do with the MS it just feels like the drug” (Patient 023 - melatonin)

*Unusual Side Effects*. A number of physical effects were perceived as unusual and unexpected by respondents and these appeared to be prevalent when taking either drug. This consisted of two isolated reports of physical sensations including headaches and dizziness when individuals began taking melatonin and one report of a strange taste while taking the placebo. The most commonly discussed side effect however was vivid dreams as four participants reported this experience while taking melatonin and two while taking the placebo.

“I had some very strange sort of headaches, it was almost like pins and needles for a couple of days when I first started taking them like sort of currents almost in my head and then when I was coming off for a couple of days of not taking them I sort of had just this funny head” (Patient 004 -melatonin)

“The first two nights I had very vivid dreams I remember that… yeah I do talk anyway in my sleep but I was really talkative apparently the first two nights I remember vaguely talking to my husband” (Patient 029 - placebo)

“I was worse. I felt I was sleeping worse, had more weird dreams and less energy from the second tablet than normal” (Patient 021 – placebo)

## 4. Discussion

Findings from this qualitative exploration on the experience of melatonin for the treatment of nocturia for people with MS revealed a number of effects. Firstly, both melatonin and matched placebo resulted in a perceived reduction of nocturia for some. This was thought to have improved the quality of sleep experienced by participants. Consequently, participants recalled feeling much more energised during the day as they reported better cognitive, psychological and physical functioning. Such improvements were mostly associated with the administration of melatonin. However, some participants also revealed a positive experience while taking the placebo. For these particular participants, the experience of sleep was subjectively better than it had been prior to trial participation, which was thought to have contributed to a feeling of having more energy during the day. However, for some participants both drugs were thought to have caused severe fatigue and tiredness, which ultimately hindered quality of life. In such situations, a reduction in nocturnal voiding was unlikely to be viewed as a beneficial improvement and so it appeared that participants were far keener to reduce their daily fatigue by improving their sleep than target nocturia. These findings were particularly important as respondents had felt that nocturia played a key role in increasing levels of fatigue and tiredness.

Aspects of this qualitative study ostensibly support previous findings by Obayashi et al. [12] and Drake et al. [13] which provided significant evidence of a link between endogenous melatonin secretion and nocturia as well as an association between exogenous melatonin and nocturia related bother in non-MS populations. The narrative accounts of participants within this study similarly suggested that melatonin was effective in reducing nocturnal voiding and any related bother. However, it also revealed that for some a placebo had been similarly influential on perceived improvements. Furthermore, participants did ultimately document adverse effects that were thought to be related to melatonin which increased levels of bother. The participants who felt that the drug had resulted in increased tiredness and fatigue stressed that their quality of life had reduced along with their nocturia. This is particularly significant, as it highlighted that participants were reluctant to view the intervention as positive if it increased elements of fatigue. This supported previous findings [16] that had recognised fatigue as the most bothersome symptom for individuals with MS; a point that was repeatedly emphasised throughout these interviews.

The findings from this qualitative study also provided more depth to the main trial findings which ultimately found that a low dose of melatonin did not achieve significant reduction in nocturia severity. It appeared that the way participants perceived their nocturia was important, as was their perspective on the level to which the symptom interfered with their lives. Those participants who continued to wake to pass urine but were able to return to sleep, perceived nocturia as a mild inconvenience, as other elements of their life were not affected. Similarly, those who reported a reduction in nocturia alongside a daily feeling of drowsiness noted that nocturia was again insignificant compared to their fatigue. This appeared to suggest that, although nocturia was problematic for this cohort, their key concern was the ability to experience deep sleep. This concern had also become apparent through discussions regarding the impact of nocturia, as all participants noted that it was their ability to sleep that appeared to have the most impact on their daily lives.

The present study is novel as it incorporates qualitative research methods into a largely quantitative drug trial, which ultimately adds more depth and insight into nocturia in MS. This is particularly crucial in health research, as it could unveil information that might not be retrieved through quantitative assessments alone. Participants' perceptions towards improved nocturia yet increased fatigue, for example, revealed the importance of eliciting patient centred, open-ended responses when attempting to investigate certain research objectives. Without the inclusion of this qualitative exploration, there could be a risk of failing to understand issues that were specific to this particular cohort, such as dissatisfaction or decreased quality of life even when nocturia had essentially improved according to the patient's perspective. Indeed, Lewin et al. [17] noted that using qualitative methods in randomised controlled trials could significantly expand our understanding of the efficacy of different interventions. Moreover, the current study also incorporated the views of partners, albeit to a limited extent, a perspective that is largely missed in exploratory studies on nocturia. In doing so, this qualitative research specifically revealed the interpersonal effect that nocturia and fatigue could have on the lives of those with MS. Furthermore, it provided some insight into the visible impact of nocturia, fatigue, and potential treatments by providing details on the effects that were observed by partners.

It is, however, recognised that this method is not without limitations. Although the use of qualitative, semistructured interviews provides a rich exploration, they also rely on participant recollections, which may be imperfect. In fact, some interviewees did disclose that they suffered from memory problems as a consequence of their MS, which could have affected the elements of their experience that they chose to share. Furthermore, participants had stressed that MS was an unpredictable condition and the changes that they experienced during the trial may well have been due to their fluctuating MS rather than the trial itself. It is acknowledged that the qualitative study explored participants' perceptions on the intervention following the final visit. To fully explore the effect of treatment, it may have been beneficial to explore perceptions towards change following each phase of the trial, which might have elicited more accurate discussions.

## 5. Conclusion

This paper presents the first qualitative exploration of using melatonin for the treatment of nocturia in individuals with MS. In doing so it sought to investigate participants' perceptions of living with nocturia as well as the effectiveness of melatonin as a potential treatment for nocturia. In summary, the interview data provided an insight into the lived experience of nocturia and found that alleviating fatigue is a key concern for people with MS who also experience nocturia. Participants reported symptomatic changes when taking either melatonin or a placebo. For some, melatonin may improve sleep, thus reducing fatigue during the day. Melatonin and placebo were also reported to have increased feelings of fatigue among some participants. These findings could therefore have many implications for practice, as these personal perspectives have revealed that melatonin may not be an appropriate treatment for all with nocturia but may offer benefits for some. Of greater benefit may be the increased understanding of the interplay between fatigue and nocturia associated with MS that could be helpful in informing improved evaluation of nocturia and its impact among individuals with these symptoms in the future.

## Figures and Tables

**Box 1:**
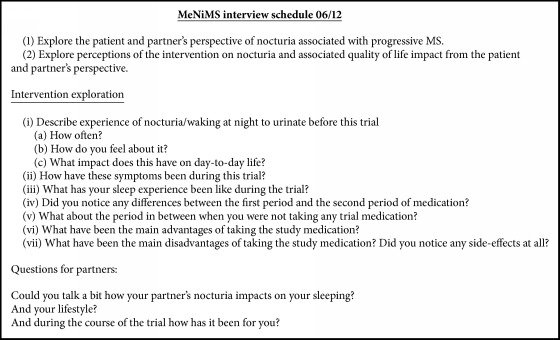
Topic guide for qualitative interviews.

**Table 1:** Demographic details for qualitative study participants.

**Variable **	**Values**

**Gender**	

Male	7

Female	10

**Age, y**	

Range	39-67

Mean	58

**Ethnicity (n)**	

White	15

No response	2

**Employment Status (n)**	

Working	5

Not working	3

No response	9

**Education (n)**	

Left school at 16 or younger	2

Left school at 17 or 18	1

College or further education	4

Graduated from university	6

No response	4

**Type of MS (n)**	

Secondary Progressive MS	9

Primary Progressive MS	2

Relapsing Remitting MS	6

## Data Availability

The interview data used to support the findings of this study have not been made available due to the ethical restrictions and consent provided by participants.
